# Editorial: Regulation of Cellular Reprogramming for Post-stroke Tissue Regeneration: Bridging a Gap Between Basic Research and Clinical Application

**DOI:** 10.3389/fcell.2021.793900

**Published:** 2021-12-14

**Authors:** Jing Wang, Cindi M. Morshead, Gong Chen, Wen Li

**Affiliations:** ^1^ Regenerative Medicine Program, Ottawa Hospital Research Institute, Ottawa, ON, Canada; ^2^ Department of Cellular and Molecular Medicine, Faculty of Medicine, University of Ottawa Brain and Mind Research Institute, University of Ottawa, Ottawa, ON, Canada; ^3^ Department of Surgery, Division of Anatomy, Temerty Faculty of Medicine, University of Toronto, Toronto, ON, Canada; ^4^ Donnelly Centre, University of Toronto, Toronto, ON, Canada; ^5^ Institute of Biomedical Engineering, University of Toronto, Toronto, ON, Canada; ^6^ Guangdong-HongKong-Macau Institute of CNS Regeneration (GHMICR), Jinan University, Guangzhou, China

**Keywords:** post-stroke regeneration, iPSCs, *in vivo* direct neuronal reprogramming, functional integration, immunomodulation

Stroke is a leading cause of death and disability world-wide. Stroke patients often live with long-term motor and cognitive impairments. There is currently no effective treatment to reverse or significantly improve these neurological outcomes. The development of cellular reprogramming technology has the potential to provide novel cell-based strategies to treat stroke-related brain injury and dysfunction. Building on well described tenets of developmental biology, the landmark discovery of induced pluripotent stem cells using defined transcription factors has since been advanced to allow the direct reprogramming of one somatic cell type to another to generate cells lost to injury or disease. This research topic explores avenues to bridge the gap between basic research and clinical application as it relates to identifying strategies that utilize cellular reprogramming for post-stroke tissue repair. The topic builds on recent advances in understanding regulatory mechanisms and approaches that can successfully reprogram non-neuronal cells into neurons through a pluripotent intermediate or bypassing pluripotency to enable neural repair. We appreciate all the researchers who participated in this topic, in which five papers were published (two original research papers and three review papers). The research presented provides valuable information and insights on cellular reprogramming as a novel therapeutic option for post-stroke tissue regeneration and functional recovery, including consideration of the microenvironment and it’s impact on reprogrammed cells. A short description of these papers follows.

In the original paper authored by Ge et al., researchers used Rhesus Macaque monkeys, non-human primates, to demonstrate that overexpression of a single neural transcription factor NeuroD1 in reactive astrocytes following ischemic injury can convert them into neurons at the injury site. Following the *in vivo* astrocyte-to-neuron (AtN) conversion, the neuronal density and synaptic markers in the NeuroD1-treated injury areas were significantly increased, accompanied with increased survival of parvalbumin interneurons and reduced number of microglia and macrophages in the lesion sites. These findings suggest that direct *in vivo* cellular reprogramming strategy not only regenerates new neurons in the injured parenchyma, but also ameliorates the niche microenvironment. The dual beneficial effects derived from direct *in vivo* cellular reprogramming in the non-human primate ischemic stroke model pave the way for future clinical studies in humans.

In a review contributed by Spellicy et al., the immunomodulatory capacity of induced pluripotent stem cells (iPSCs) in the post-stroke environment is discussed. iPSCs are generated from somatic cells, such as skin fibroblasts, by ectoptic expression of four key transcription factors that are essential to pluripotency (Takahashi and Yamanaka, 2006). Following transplantation of iPSCs, it has been purported that iPSC progeny contribute new cells to repopulate damaged tissues, including neurons, which may underlie some of the therapeutic potential of iPSCs. This review discussed the substantial paracrine effect of iPSCs and their derivative cells on neuroinflammation. Specifically, they provide evidence that iPSCs, iPSC-neural progenitor cells (iPSC-NPCs), and iPSC-neuroepithelial like stem cells (iPSC-lt-NESC) can significantly modulate proinflammatory signaling and endogenous inflammatory cell populations, such as microglia phenotypes. This review provides a comprehensive examination of the mechanisms by which iPSCs mediate neuroinflammation in the post-stroke environment, as well as delineate avenues for further investigation. Understanding underlying mechanisms that mediate anti-inflammatory effects of iPSCs and their derived cells is important for determining the optimal dosing and timing of iPSC-derived neural cell transplantation to ensure we are capturing the beneficial post-stroke immune responses.

A review paper by Palma-Tortosa et al. summarized recent studies using advanced research tools, such as optogenetic control of neuronal activity and rabies virus monosynaptic tracing, to provide compelling evidence that functional integration of grafted iPSC-derived neurons occurs in the impaired brain circuitry resulting from stroke. Indeed, they argue that it is the functional integration that leads to long-term structural and functional repair following stroke. More interestingly, their own work demonstrated that *ex vivo* transplantation of human skin-derived neurons in organotypic cultures of adult human cortex can be integrated into the human lesioned neuronal circuitry (Grønning Hansen et al., 2020). This human-to-human graft study strongly supports that neuronal replacement using human skin-derived neurons, via iPSCs, is a key contributing factor for the success of stem cell therapy to treat stroke. This review paper highlights the need for understanding the cellular mechanisms that underlie transplant success, further supporting that functional integration of grafted iPSC-derived neurons into the existing circuitry in the injured brain is a key for success.

An obvious benefit of iPSCs is the ability to generate autologous neural cell lineages to repair the stroke-damaged brain (Duan et al., 2021). Similarly, direct neuronal reprogramming affords the generation of new cells without the need for non-autologous cell transplantation. Direct cellular reprogramming is an innovative approach to convert somatic cells to induced neurons (iNs) without passing through a pluripotent state. Vasan et al. gave a thorough overview of the history, accomplishments, and therapeutic potential of direct neuronal reprogramming in treating neurodegenerative diseases and brain injuries such as stroke, over the last 2 decades. It summarized and discussed the roles of many key factors, including transcription factors, microRNAs, small molecules and other growth factors in direct neuronal reprogramming by recapitulating the developmental process of the developing brain both *in vitro* and *in vivo*. This review paper described not only novel insights of the basic epigenetic mechanisms, such as histone modifications, DNA methylation and chromatin remodeling, involved in neuronal reprogramming, but also the potential applications of direct neuronal reprogramming on disease modelling and treatment of neurodegenerative diseases. Thus, direct neuronal reprogramming represents a revolutionary strategy to generate iNs, avoiding tumorigenicity of iPSC cells and technical limits related to iPSC transplantation such as invasiveness and inability to produce sufficient neuronal cells in a timely fashion.

Direct lineage reprogramming of endogenous astrocytes into neurons *in situ* has become an attractive technology to simultaneously replenish the neuronal population and reduce the glial scar following brain injury (Zhang et al., 2020), a key question is whether the newly reprogrammed neurons undergo normal development, integrate into the existing neuronal circuit, and acquire circuit appropriate functional properties. In this regard, Tang et al. used a murine ischemic visual cortex stroke model to investigate the effect of NeuroD1-mediated *in vivo* direct reprogramming on visual cortical circuit integration and functional recovery. The group used electrophysiological extracellular recordings, two-photon calcium imaging of reprogrammed cells *in vivo* and mapping the synaptic connections *ex vivo* to show that NeuroD1 reprogrammed neurons were integrated into the cortical microcircuit and acquired direct visual responses. Additionally, Tang et al. demonstrated that the reprogrammed neurons can mature over time, manifested by alterations of orientation selectivity and functional connectivity. This article presents important evidence at both the cellular and system levels, showing that the astrocyte-converted neurons *in situ* following stroke can successfully replace the lost neurons following brain injury and functionally integrate into the circuitry.

These striking findings, and important insights into the cellular mechanisms provided in this research topic, support the promise of reprogramming strategies to enhance neural repair and functional recovery of the stroke injured brain. While the recent advances of iPSC transplantation and direct neuronal reprogramming at sites of injury hold significant potential for clinical translation, supported in large part by the non-human primate stroke studies Ge et al., the gaps are still in need of filling. For reprogramming strategies *in vivo*, discerning the safe and efficient delivery route; establishing the best gene cargo that will generate region and circuit appropriate neural phenotypes and the choice of viral vectors are areas that will need to be focussed. Similarly, the source of neural cells for transplantation, the transplant location and the time of transplantation need to be clearly established. Indeed, relevant to all cell-based approaches for stroke injury repair, determining the time window for post-stroke interventions is critical for moving to clinic. With the knowledge of the enormous global socioeconomic burden that stroke places on patients, families and caregivers, it is critical to continue to focus next steps on understanding the cell basis for the promising outcomes to date in order to support the translation of these exciting approaches to advance stroke recovery.
